# Negative Outcomes Associated with Medication in Neonates on Parenteral Nutrition Therapy

**DOI:** 10.3390/pediatric15020032

**Published:** 2023-06-06

**Authors:** Evelin Nataly Vega Díaz, Aida Adriana Miranda Barros, Monica Alexandra Castelo Reyna, Dennys Tenelanda López, Irvin Tubon

**Affiliations:** 1 Agencia Nacional de Regulación, Control y Vigilancia Sanitaria (ARCSA), Quito 090112, Ecuador; 2 Grupo de Investigación de Tecnología y Atención Farmacéutica del Ecuador (GITAFEC), Escuela Superior Politécnica de Chimborazo (ESPOCH), Riobamba 060155, Ecuador; 3 College of Mechanics, School of Industrial Maitance, Escuela Superior Politécnica de Chimborazo (ESPOCH), Riobamba 060155, Ecuador; 4 School of Dentistry, Universidad Nacional de Chimborazo (UNACH), Riobamba 060110, Ecuador; 5 College of Natural Resources, Escuela Superior Politécnica de Chimborazo (ESPOCH), Riobamba 060155, Ecuador; 6 Carrera de Biotecnología, Facultad de Ciencia e Ingeniería en Alimentos y Biotecnología, Dirección de Investigación y Desarrollo, Ambato 180207, Ecuador

**Keywords:** drug-related problems, neonatology, pharmacotherapeutic follow-up

## Abstract

Objective: In Ecuador, studies on clinical daily practice problems focused on parenteral nutrition in neonates are scarce. Therefore, this research aimed to identify negative results associated with medications (NRAM) in neonates with parenteral nutrition (PN) in a third-level hospital in Ecuador. Material and methods: An observational, prospective, descriptive study was designed in the neonatology area of a tertiary-level public hospital, where, for over four months, the medical records, PN prescriptions, and pharmacy-managed databases of 78 patients were analyzed. Drug-related problems (DRPs) as possible causes of NRAM were classified through administrative, physicochemical, and clinical validation. Results: DRPs classified as follows were found: 78.81% by physicochemical, 17.62% by clinical, and 3.57% by administrative validation. The NRAM were 72% quantitatively uncertain, 16% needed, and 11% quantitatively ineffective. Conclusion: The NRAM associated with DRPs were statistically related to prematurity condition, APGAR score, PN time, and the number of medications administered, which suggests the need to create a nutritional therapy committee at the health facility.

## 1. Introduction

The negative results associated with the medication (NRAM) are presented as complications in the patient’s health manifested by clinical events or even death. Drug-related problems (DRPs) that cause these complications are those situations that, in the process of drug use, cause or may cause the appearance of a negative result associated with the medication [1,2].

Parenteral nutrition (PN), defined as the administration of macro and micronutrients through the central and/or peripheral route, is an essential therapeutic resource in newborns (NB) and crucial in preterm newborns (PNB) who present high nutritional demands and do not have adequate maturity of organs, systems, and metabolic pathways that allow the assimilation of substances by the enteral route [3,4].

As it is a frequent therapy at the hospital level, complications are common in poly-pathological and poly-medicated patients who are exposed to the accumulation of drugs in the circuits of the different medical devices used in PN, representing a risk of physicochemical incompatibility. Other clinical complications derive from the medical indication that are usually associated with difficulties in caloric adjustment, alterations related to intake, and the requirement of macro and micro molecules [5,6]. The in-line filtration associated with inappropriate multi-lumen systems and the above drawbacks prevents PN from fulfilling the nutritional therapeutic objective [7,8,9,10].

PN is defined as a high-risk medication requiring special management measures. USA reports revealed that only 58% of organizations have procedures focused on preventing errors and harm in patients with PN. There is a general deficit in the processes related to the prescription, validation, composition, preparation, dispensing, and administration of PN. In addition, 23% of the establishments do not have a pharmacist dedicated to the administrative, physicochemical, and clinical validation of PN orders [11,12,13,14].

Therefore, this research aims to show an overview of the main DRPs associated with the clinical practice of PN in neonatology, as well as propose strategies to identify, resolve, and prevent NRAM.

## 2. Materials and Methods

### 2.1. Sample

A prospective, observational, descriptive study was conducted in the neonatology department of a tertiary public hospital over a four-month period. The study was overseen by a specialist physician responsible for nutritional assessment and the prescription of caloric intake. The pharmacist was responsible for the administrative, physicochemical, and clinical validation of the parenteral nutrition (PN). Finally, the nursing staff administered PN and provided necessary care. Convenience sampling was used to select 78 patients who received PN (total, partial, and cycled) and complementary enteral nutrition (CEN). Patients who did not receive PN or only received enteral nutrition (EN) were excluded.

### 2.2. Data Collection Tools

General clinical information such as gestational age (GA), birth weight (BW), APGAR score, sex, delivery type, daily review medical records, laboratory test reports and PN prescriptions were recorded using a Microsoft excel database. The study used the parenteral nutrition guidelines AS-PEN/ESPGHAN/ESPEN/ESPR/CSPEN/NICE to analyze caloric-nutritional intake and pathologies associated with therapy. The DADER pharmaco-therapeutic follow-up method was employed as a data collection resource.

### 2.3. NRAMs Identification

The study identified drug-related problems (DRPs) as causal agents of negative results associated with the medication (NRAM different non-routine adverse events, NRAMs) through three types of validations managed by the pharmacy: administrative, physicochemical, and clinical. Administrative validation involved a qualitative and comprehensive review of the prescription received to ensure the integrity and clarity of patient data, nutritional requirements, and prescriber data. Physicochemical validation focused on calculating the mixture’s composition, stability through analytical relationships, and mixing order. Clinical validation assessed and analyzed the patient’s needs, safety, and effectiveness of pharmacotherapy. The possible NRAMs were classified according to the criteria of necessity, safety, and effectiveness established according to the third consensus of Granada-2007. The study conducted a daily review of medical records, laboratory test reports, PN prescriptions sent to the pharmacy, and databases managed in the service to collect information. In addition, gestational age (GA), birth weight (BW), APGAR score, sex, and delivery type were recorded. Reports on hepatic, renal, hematic, respiratory, electrolytes, gasometric, and metabolic profiles were also analyzed.

The protocol was evaluated and approved by the Human Research Ethics Committee of the Pontificia Universidad Católica del Ecuador (CEISH-PUCE), with registration number PV-03-202.2.4.

### 2.4. Statistical Analysis

Data analysis was performed using R version 4.0.3 (R Core Team, 2020) with the following packages: ggplot2 version 3.3.2 for data visualization and dplyr version 1.0.2 for data manipulation.

General information was detailed using descriptive statistics. The R statistical program estimated the relationship between DRPs and NRAM with Fisher’s exact test (*p* ˂ 0.05). Finally, the possible statistical association of NRAM with factors specific to the patient was carried out using multivariate statistical significance analysis risk ratio, RR).

## 3. Results

### 3.1. Population Characteristics

Information on 78 neonates was recorded (Table 1) with a 51% male and 49% female distribution. The data showed patients admitted with low birth weight (LBW) (40%), very low birth weight (VLBW) (34%), and preterm newborns (PNB) (87%) subclassified as late 44%, moderate 24%, severe 10%, and extreme 9%.

A total of 920 PN prescriptions were received, where “prematurity” predominated as a baseline diagnosis, and 36% of neonates received PN for seven days, 32% up to 14 days, 18% up to 21 days, 13% up to 28 days, and 1% for more than 28 days.

In the Neonatal Intensive Care Unit (NICU), multi-pathological poly-medicated patients with complex drug treatment (approximately 11 drugs/day) and patients in intermediate care with an approximate average of 2 drugs/day, and PN only in projected weight gain were found. The mixtures prepared were 3 in 1 (dextrose, lipids, and amino acids in the same PN bag 50%), 2 in 1 + separate lipids (dextrose and amino acids in a PN bag, while lipids were in another 45% infusion line), and 2 in 1 (dextrose and amino acids in a bag of PN 5%). A 22% mortality rate was estimated.

### 3.2. Drug-Related Problems (DRPs)

The analysis of the 920 prescriptions showed approximately 7.6 DRPs for each prescription (Table 2), classified as 78.81% by physicochemical validation, 17.62% by clinical validation, and 3.57% by administrative validation.

### 3.3. Types of NRAM

Table 3 shows 61 NRAM classified as 72% due to TYPE VI quantitative insecurity where the patient received a concentration of the nutritional component and/or duration greater than necessary, 16% TYPE I need due to non-administration of the necessary nutritional component, and 11% quantitative ineffectiveness of TYPE IV where the patient received a concentration of the nutritional component less than required.

Fisher’s exact test suggests a statistical association (*p <* 0.05) of dependence between the DRPs and NRAM: “Necessary phosphorus not available” and “alkaline phosphatase elevation”; “Necessary vitamins not available” and “Lactic acidosis”; “Lipid level > necessary” and “cholestasis”; “Carbohydrate rate > necessary” and “hyperglycemia”. In addition, the results suggest a risk relationship between “Lipid levels > the necessary” and “cholestasis”, RR: 3.2 (95% CI (Confidence interval) 1.720–6.109).

For the parameter “Carbohydrate rate > necessary” and “hyperglycemia”, the relationship is similar RR: 2.8 (95% CI 1.203–6.430).

The multivariate statistical significance analysis (*p <* 0.05) (Table 4) indicates that the condition of prematurity (PNB) significantly influences (*p* = 0.031) the NRAM “hyperglycemia”, as well as for the NRAM “cholestasis” (*p* = 0.029).

On the other hand, the APGAR score influences “lactic acidosis” (*p* = 0.003) NRAM, as well as the time and number of drugs administered during PN on the “elevation of alkaline phosphatase” with *p* = 0.037 and *p* = 0.014, respectively.

## 4. Discussion

The DRPs found were divided into administrative (3.57%), physicochemical (78.81%), and clinical validation (17.62%). Among the possible causes of NRAMs found by administrative validation, the majority (25.6%) focused on the weight of the NB declared in the evolution sheet (ES), since the orders arrived at the pharmacy with different or inconsistent information. They constitute a source of risk to triggering fluid and electrolyte imbalance and deficit or excess nutrient intake associated with exchanging in training or not providing nutritional therapy [15,16].

On the other hand, due to a lack of familiarity with the input, an error was found in calculating the caloric intake (16.69%) through physicochemical validation. The prescriber took 4 Kcal/g as a bibliographic reference to estimate the volume of glucose infusion (VGI). However, the input available in the unit declares 3.4 Kcal/g, which is a similar situation to the contribution of lipids. Consequently, 81.92% of the mixtures were prepared with a deficit of up to 13.8 Kcal/kg/day and 6.47%, with a deficit of up to 28 Kcal/kg/day [14,17].

Similarly, when the NB progressively tolerated enteral nutrition (EN), the daily concentration of PN components was reduced, decreasing the caloric rate to balance energy intake. However, the record on the evolution sheet did not change. Theoretically, 1.34% of the prescriptions were administered with a deficit of up to 72.4 Kcal/kg/day.

The “Rate of components or Kcal > than necessary” was frequent in patients who tolerated adequate volumes of EN (LPN (L): 140 to 150 mL/kg/day and TN: 120 to 140 mL/kg/day) [18,19,20]. Only the readjustment in the volume of PN to be prepared was evidenced and not in the concentration of nutrients that occupied the mixture, which could cause the patient to receive an intake more significant than the oxidation limit level [21].

As regards macronutrients, the DRP “amino acid rate > the necessary” (4 g/kg/day), (6.30%) was evidenced. This could probably be associated with NRAM of metabolic acidosis, azotemia, and elevated urea nitrogen in blood (BUN), triggering hyperammonemia in neurocognitive developmental imbalance, cholestasis due to prolonged administration, and electrolyte imbalance in the first days [22,23]. On the other hand, the “Lipid rate > that required” (3 mg/kg/day) (5.93%) could increase the risk of NRAMs, such as hypertriglyceridemia, liver enzyme imbalance, lipid overload syndrome, and displacement of drug plasma protein binding [24,25].

The DRP “Carbohydrate rate > necessary” (1.96%) (2 mg/kg/min) denotes that the suggested maximum oxidation rate was exceeded and can cause hyperglycemia (with water retention), risk of cardiotoxicity, hepatic steatosis, hypertriglyceridemia, increased CO_2_ production, and other respiratory problems, associated or not, and mechanical ventilation could increase [12,26,27]. Although other pediatric PN guidelines suggest 16 mg/kg/min (23 g/kg/day) as a maximum intake for LPN (L) and 13 mg/kg/min (18 g/kg/day) in TN, the health unit handles 12.5 mg/kg/min as the maximum limit [22].

The “Electrolyte rate > that required (summed as mEq/kg/day of chlorine)” of 3.70% indicates that the administration of the micronutrients sodium chloride and potassium chloride exceeded the suggested limits of 6.5 mEq/kg/day (suggested range: NaCl + KCl = 1–2 mmol/kg/day), increasing the risk of “hyperchloremic acidosis”, imbalances associated with intraventricular hemorrhage (IVH), and other comorbidities in newborns [28].

The DRP “Fluid rate > the necessary” (0.04%) (LNP (L) is max 180 mL/kg/day; PNB max 160 mL/kg/day) is reported due to errors in the prescription or adjustment of liquid; representing a greater risk of NRAM such as pulmonary broncho dysplasia, intraventricular hemorrhage, sepsis, metabolic acidosis, lactic acidosis, respiratory ischemia, heart failure, and generalized deterioration of the clinical picture [28,29].

The “Calorie rate < the necessary” (5.82%) refers to the prescription of energy intake below the ranges recommended by the EPSGHAN (RNPT in stable phase: 90–120 Kcal/kg/day; PNB in stable phase: 75–85 Kcal/kg/day) [19].

Regarding the DRP “Phosphorus required not available” (16.69%), phosphorus was not administered to patients during the entire therapy due to insufficient input in the health unit. This inconvenience increases the risk of developing refeeding syndrome (RS) due to the mobilization of minerals from the “reserves” (bones and kidneys) to support anabolic processes [30]. In addition, studies warn that insufficient supplementation of this mineral would cause the release of phosphorus from the bone reserve with the simultaneous mobilization of calcium, causing in the long term the metabolic bone disease (MBD) RNAM with hypocalcemia less than 2.2 mmol/L, hypophosphatemia less than 4.5 mg/dl or 1.45 mmol/L, and the elevation of alkaline phosphatase more significant than 500 IU/L (visible at 2 weeks of postnatal age); the results were corroborated using imaging tests [31,32].

In turn, uncontrolled chronic hypophosphatemia can cause respiratory and myocardial failure due to ATP depletion of the myocyte, rhabdomyolysis, anemia, hemolysis, seizures, and metabolic acidosis [33].

The DRP “Necessary vitamins not available” (16.69%) indicates an acquisition problem related to administrative management and/or market availability, which leads to prescribing several vitamins that cover essential biological functions. The deficiency, mainly of thiamine pyrophosphate, may be related to damage to the oxidative metabolism of carbohydrates, proteins, and fatty acids, contributing to the appearance of lactic acidosis [34].

Clinical validation made it possible to describe the DRP “Lipid infusion rate > the recommended 1.7 mg/kg/min (0.10 g/kg/h) (2.11%)”, which refers to the rate of infusion higher than the estimated limits for the oxidation capacity, increasing the risk of lipid overload with consequent pulmonary edema. The DRP “Lipid infusion rate < the recommended 1.2 mg/kg/min (0.07 g/kg/h)” (7.55%) states that the objective nutritional guideline would not be being met in the patient [35].

The DRP “Toxicity risk associated with packaging material” (28.25%) refers to the risk of aluminum contamination for using calcium gluconate in glass ampoules as components of PN. The Food and Drug Administration (FDA) suggests that the declared aluminum concentration should not exceed 25 mcg/L and for no reason should exceed 5 mcg/kg/day of aluminum in the therapy of the patient with PN [10]. Otherwise, the NB, especially LPN(L) with PN for more than 10 days, could suffer damage in the neurocognitive development, bone mineralization problems, long-term renal failure, cholestasis, and liver damage reported in other studies [36,37].

The incompatibility in “Y” (2.11%) mainly describes the association of PN with dopamine and/or dobutamine, for which the pharmacotherapeutic follow-up service (SFT) of the health unit advises that joint infusion with these drugs for 24 h can cause a loss of up to 10% of the active concentration and decreased effectiveness. The reaction could be explained by the pH of the lipid emulsion; thus, for PN 2 in 1, no incompatibility is reported [38].

Possible PN drug interactions (0.24%) were also determined. Linezolid, a licosamide with MAOI action, can interact with tyrosine, phenylalanine (dopamine precursors), and tryptophan (serotonin precursor), causing a difficult-to-manage dopaminergic or serotonin syndrome, especially in critically ill patients who maintain a dopamine infusion pump. The fluconazole/PN association may contribute to the elevation of liver markers, increasing the risk of cholestasis or complications of a pre-existing condition [25,39,40].

The limitations for detecting NRAM refer to problems of acquisition and availability of reagents and/or recommended markers necessary for the follow-up of patients with nutritional therapy, such as the case of phosphorus and BUN. Although there are studies focused on DRPs as causes of NRAM, these are scarce in parenteral nutrition, so the research presented here describes several novel or unconventional DRPs in pharmaceutical practice [7,9,11,41,42].

## 5. Conclusions

In conclusion, although there are studies focused on DRPs as causes of NRAM, these are scarce in parenteral nutrition, so this research describes several novel or unconventional DRPs in pharmaceutical practice. The information provided in this research can serve as a precedent for creating hospital nutritional therapy committees focused on the individualized analysis of nutritional needs that allow identifying, resolving, reducing, and preventing risks associated with PN.

## Figures and Tables

**Table 1:** General characteristics of the study population.

Description	Average ± SD	Min–Max.	N	%
**Gender**				
Male	-	-	40	51
Female	-	-	38	49
**Type of birth**				
Cesarean	-	-	69	88
Normal	-	-	9	12
**Weight**				
Birth weight	1.640 ± 0.644	0.670–3.845		
Adequate birth weight (ABW)	2.955 ± 0.468	2.506–3.845	10	13
Low birth weight (LBW)	1.774 ± 0.232	1.520–2.440	31	40
Very low birth weight (VLBW)	1.294 ± 0.165	1.00–1.480	27	34
Extremely low birth weight (ExLBW)	0.832 ± 0.100	0.670–0.960	10	13
**Age**				
Gestational age	33.000 ± 3.100	25.520–40.401		
Term newborn (TN)	37.812 ± 1.250	36.421–40.401	10	13
Late preterm newborn LPN (L)	34.100 ± 0.921	33.100–36.405	34	44
Moderate preterm newborn MPN (M)	31.901 ± 0.805	30.212–33.001	19	24
Severe preterm newborn SPN (Sev)	29.305 ± 0.412	29.005–30.002	8	10
Extremely preterm newborn EPN (Ext)	26.704 ± 0.912	25.013–28.025	7	9
**Score APGAR**				
Minute 1	7.001 ± 1.805	1.001–10.002		
(a) 7–10	7.950 ± 0.651	7.002–8.012	59	76
(b) 4–6	4.932 ± 0.832	4.043–6.023	14	18
(c) NV-3	1.753 ± 0.961	NV–3.0	5	6
Minute 5	9.001 ± 1.305	1.001–10.015		
(a) 7–10	8.812 ± 0.412	7.023–10.012	72	92
(b) 4–6	6.005 ± 0.000	6.001	3	4
(c) NV-3	2.012 ± 1.405	NV–3.001	3	4
**Nutritional therapy**				
Time of nutritional therapy administered (days)	12.001 ± 7.705	1.001–30.049		
(a) >28	23.601 ± 1.323	31.001	1	1
(b) 21–28	24.701 ± 2.105	22.013–28.011	10	13
(c) 14–21	18.071 ± 1.701	15.001–21.002	14	18
(d) 7–14	10.962 ± 1.932	8.000–14.005	25	32
(e) 1–7	3.961 ± 2.105	1.000–7.015	28	36
**Mix type**			920	100
2 in 1	-	-	43	5
2 in 1 + lipids apart	-	-	418	45
3 in 1	-	-	459	50
**Drugs**				
Number of drugs administered during nutritional therapy (drugs/day)	5.001 ± 2.122	1.501–10.561	-	-
**Mortality**				
Yes	-	-	17	22
No			61	78

NV: no value; adequate birth weight (ABW): heavier than 2500 g; low birth weight (LBW): weight < 2500–1501 g; very low birth weight (VLBW): weight < 1500–1001 g; extremely low birth weight (ExLBW): weight < 1000 g; term newborn (TN): gestational age 37–41 weeks; late preterm newborn LPN (L): gestational age 34–36.6 weeks; moderate preterm newborn MPN (M): gestational age 31–≤33 weeks; severe preterm newborn SPN (Sev): gestational age 29–≤30 weeks; extremely preterm newborn EPN (Ext): gestational age ≤ 28 weeks; APGAR: test that evaluates appearance (skin color), pulse (heart rate), irritability (Grimace in English), activity (muscular tone), respiration (rate and respiratory effort); >7 continue with mother until minute assessment 5 y <7 transfer to the care area for the evaluation and stabilization; mix type 2 in 1: includes amino acids and carbohydrates in the same bag of PN; 2 in 1 + separate lipids: includes amino acids and carbohydrates in the same PN bag and lipids administered in Y; 3 in 1: includes lipids, amino acids, and carbohydrates in the same bag of PN.

**Table 2:** Drug-related problems (DRPs) as administrative, physicochemical, and clinical validation.

**DRPs**	**n**	**%** **Validation**	**% Total**
**Administrative Validation**	250	100	3.57
Error or absence in the patient data	64	25.60	
Evidence of amendment	29	11.62	
Typing error of the components in the prescription Typing error in the calories declared in the evolution sheet	47	18.82	
Typing error in the rate of micronutrients declared in the evolution sheet	38	15.20	
Typing error in the total volume to prepare the PN and/or the infusion rate declared in the progress sheet	19	7.62	
Typing error in the dextrose rate declared in the evolution sheet	18	7.21	
Typing error in the lipid rate declared in the evolution sheet	15	6.00	
Typing error in the declared amino acid ratio in the evolution sheet	13	5.21	
Duplication of prescription sheets	3	1.23	
Wrong prescription for the patient	3	1.22	
Omission of the prescription	1	0.42	
**Physicochemical validation**	5512	100	78.81
Calculation error of caloric intake and/or declared value in the clinical history	920	16.69	
Necessary vitamins not available	920	16.69	
Phosphorus required not available	920	16.69	
Filters 1.2 µ not available	877	15.91	
Filters 0.22 µ not available	461	8.36	
Amino acid rate > necessary	347	6.30	
Lipid rate > to necessary	327	5.93	
Calorie rate < to necessary	321	5.82	
Electrolyte rate > that required (summed as mEq/kg/day of chlorine)	204	3.70	
Carbohydrate rate > necessary	108	1.96	
Lipid rate < to necessary	70	1.27	
Calorie rate > the necessary	18	0.33	
Carbohydrate rate < to necessary	17	0.31	
Fluid rate > to necessary	2	0.04	
**Clinic Validation**	1232	100	17.62
Toxicity risk associated with packaging material.	348	28.25	
Kcal/gN ratio < (100–130)	169	13.72	
Phosphorus analysis not available	129	10.47	
Magnesium analysis not available	129	10.47	
BUN test not performed	129	10.47	
Lipid infusion rate <recommended (0.35 mL/kg/h)	93	7.55	
Kcal/gN ratio < (150–200)	88	7.14	
Lipid infusion rate > recommended (0.51 mL/kg/h)	26	2.11	
PN-drug incompatibility (dopamine and/or dobutamine)	26	2.11	
ALT analysis not available	23	1.87	
AST analysis not available	23	1.87	
Direct bilirubin analysis not available	23	1.87	
Alkaline phosphatase test not available	23	1.87	
PN-drug interaction	3	0.24	

DRP: Drug-related Problems; PN: parenteral nutrition; BUN: blood urea nitrogen; AST: Aspartate Aminotransferase; ALT: Alanine aminotransferase.

**Table 3:** NRAM classification, statistical association (*p <* 0.05), and risk relationship with DRPs.

Classification of NRAM Granada 2007 (%)	Criteria	NRAM	n	%	DRPs	*p*-Value	RR (CI 95%)
NEED (16)	(I): Untreated health problem	Alkaline phosphatase elevation	7	11	Phosphorus needed not available	˂0.001	0.4 (0.141–0.648)
		Lactic acidosis	3	5	Necessary vitamins not available	0.001	0.2 (0.073–0.681)
EFFECTIVITY (11)	(IV): Ineffective quantitative	Hypoalbuminemia	5	8	Relation Kcal/gN < (100–130)	0.570	1.4 (0.537–3.396)
		Hypoglycemia	2	3	Carbohydrate rate < necessary	0.127	4.5 (0.768–26.405)
SECURITY (72)	(VI): Insecurity quantitative	Cholestasis	12	20	Lipid level > to necessary	0.001	3.2 (1.720–6.109)
		Hyperchloremia	9	15	Electrolyte rate > that required (summed as mEq/kg/day of chlorine)	0.845	1.0 (0.548–2.005)
		Elevated liver enzymes	7	11	Lipid level > to necessary	0.165	1.9 (0.855–4.183)
		Metabolic acidosis	6	10	Amino acid rate > necessary	0.432	1.4 (0.605–3.227)
		Hyperglucemia	7	11	Carbohydrate rate > necessary	0.024	2.8 (1.203–6.430)
		Hypertriglyceridemia	2	3	Lipid level > to necessary	0.083	6.8 (0.969–47.067)
		Hypercalcemia	1	2	Calcium level > necessary	0.340	3.4 (0.311–36.684)

NRAM: negative result associated with medication. DRPs: drug-related problems. (I): Untreated health problem: the patient suffers from a health problem associated with not receiving the necessary medication. (IV): Quantitative ineffectiveness: the patient suffers from a health problem associated with a quantitative ineffectiveness of the medication. (VI): Quantitative uncertainty: the patient suffers from a health problem related to quantitative uncertainty about a drug. Alkaline phosphatase elevation: >500 UI/L. Lactic acidosis: anion gap ({Na+}–{Cl^−^ + CO_3_H^−^} > 14 and lactic acid > 2.1 mmol/L. Hypoalbuminemia: albumin in the blood < 30 g/L. Hypoglycemia: normal glucose range 40–45 mg/dL. Cholestasis: direct bilirubin (DB) greater than 2 mg/dL or greater than 20% of total bilirubin (BT) when BT is higher than 5 mg/dL. Hyperchloremia: normal range 100–109 mmol/L, elevated > 109 mmol/L. Elevated liver enzymes: alanine aminotransferase (ALT) normal range 0–35 IU, aspartate aminotransferase normal range 0–35 IU, metabolic acidosis: anion gap ({Na+}–{Cl^−^ + CO_3_H^−^} > 14. Hyperglycemia: glucose concentration > 145 mg/dL. Hypertriglyceridemia: normal range 150–200 mg/dL, elevated > 200 mg/dL. Hypercalcemia: normal range 1.12–1.32 mmol/L, elevated 1.32 mmol/L. CI: confidence interval at 95%.

**Table 4:** Multivariate analysis of negative results associated with medication (*p <* 0.05).

Factors	Alkaline Phosphatase Elevation	Lactic Acidosis	Cholestasis	Hyperglycemia
Birth weight	0.393	0.197	0.442	0.075
**Gestational age (GA)**				
PNB	0.967	0.188	0.780	0.031 *
TN	0.519	0.700	0.029 *	0.764
**APGAR** **assessment**				
Apgar 1′	0.464	0.183	0.788	0.696
Apgar 5′	0.776	0.030 *	0.724	0.629
**Time of nutritional therapy administered (days)**				
<14 days	0.882	0.785	0.785	0.427
>14 days	0.037 *	0.337	0.966	0.056
Number of drugs administered during nutritional therapy (drug/day)	0.014 *	0.214	0.457	0.766

* *p* < 0.05. PNB: preterm newborn; TN: term newborn.

## Data Availability

Data were collected on an Excel spreadsheet for analysis, and results presented in this paper were shared with the author team.

## Abbreviation

NRAMs	negative results associated with medications
NRAM	Negative result associated with medication
PN	parenteral nutrition
DRPs	drug-related problems
NB	newborns
PNB	preterm newborns
CEN	complementary enteral nutrition
EN	enteral nutrition
GA	gestational age
BW	birth weight
APGAR	appearance, pulse, grimace, activity, respiration.
CEISH-PUCE	Human Research Ethics Committee of the Pontificia Universidad Católica del Ecuador
VLBW	very low birth weight
NICU	neonatal intensive care unit
TN	term newborn
LPN (T)	late preterm newborn
MPN (M)	moderate preterm newborn
SPN (Sev)	severe preterm newborn
EPN (Ext)	extremely preterm newborn
ABW	adequate birth weight
LBW	low birth weight
ExLBW	extremely low birth weight
DB	direct bilirubin
BT	total bilirubin
ALT	alanine aminotransferase
GA	gestational age
ES	evolution sheet
VGI	volume of glucose infusion
EUN	elevated urea nitrogen
IVH	intraventricular hemorrhage
NPO	nothing by mouth
MCT	medium chain triglycerides
SIRS	systemic inflammatory response syndrome
FDA	Food and Drug Administration
SFT	follow-up service
ESPGHAN	European Society for Pediatric Gastroenterology Hepatology and Nutrition
